# Cardiovascular and glucose-lowering medication use among older adults: results from 9-year follow-up of the FINGER trial

**DOI:** 10.1007/s41999-025-01354-1

**Published:** 2025-12-03

**Authors:** Maria Sääskilahti, Emma Aarnio, Esko Levälahti, Jenni Lehtisalo, Miia Kivipelto, Timo Strandberg, Riitta Antikainen, Hilkka Soininen, Tiina Laatikainen, Jaakko Tuomilehto, Alina Solomon, Francesca Mangialasche, Tiia Ngandu

**Affiliations:** 1 Department of Public Health, Lifestyles and Living Environments, Finnish Institute for Health and Welfare, 00271 Helsinki, Finland; 2 School of Pharmacy, University of Eastern Finland, 70211 Kuopio, Finland; 3 Institute of Public Health and Clinical Nutrition, University of Eastern Finland, Kuopio, Finland; 4 Division of Clinical Geriatrics, Center for Alzheimer Research, Department of Neurobiology, Care Sciences and Society, Karolinska Institutet, 171 64 Solna, Stockholm, Sweden; 5 The Ageing Epidemiology Research Unit, School of Public Health, Imperial College London, London, UK; 6 University of Helsinki and Helsinki University Hospital, Helsinki, Finland; 7 Research Unit of Population Health/Geriatrics, University of Oulu, 90014 Oulu, Finland; 8 Medical Research Center Oulu, Oulu University Hospital, Oulu, Finland; 9 Department of Clinical Medicine/Neurology, University of Eastern Finland, 70211 Kuopio, Finland; 10 Department of Neurology, Kuopio University Hospital, Kuopio, Finland; 11 Wellbeing Services County of North Karelia (Siun Sote), Joensuu, Finland; 12 South Ostrobothnia Central Hospital, Seinäjoki, Finland; 13 Department of Public Health, University of Helsinki, Helsinki, Finland; 14 Theme Inflammation and Aging, Medical Unit Aging, Karolinska University Hospital, Stockholm, Sweden

**Keywords:** Cardiovascular, Glucose-lowering, Medication use, Register, Older people

## Abstract

**Purpose:**

This study aimed to investigate the use of antihypertensive, lipid-lowering, antithrombotic, and glucose-lowering medication among the FINGER (Finnish Geriatric Intervention Study to Prevent Cognitive Impairment and Disability) population during a 9-year follow-up. Also, the effects of the FINGER intervention on and differences between sexes and age groups in medication use were studied.

**Methods:**

Medication data for FINGER participants were retrieved from a national register annually, starting from the study baseline. FINGER was a two-year randomized controlled trial for older adults (*n* = 1259) at risk of cognitive impairment, who were randomly allocated to a multidomain lifestyle intervention or a control group. The intervention aimed at enhancing healthy lifestyle and to manage and monitor cardiovascular and metabolic risk factors. Generalized Estimation Equations were used to assess longitudinal changes in medication use.

**Results:**

Cardiovascular and glucose-lowering medication use was common and increased over time among FINGER participants. The prevalences of medication use did not differ between the intervention and control groups. The use of cardiovascular and glucose-lowering medication was fairly similar between men and women. Older participants used more cardiovascular medication compared to younger participants throughout the follow-up.

**Conclusion:**

The increasing use of cardiovascular and glucose-lowering medication in this older population at risk of cognitive impairment reflects increasing cardiovascular and diabetes morbidity, management of these risks and diseases through medication, and possible changes in medical practices. Before drawing further conclusions about the effect of the intervention on medication use, its effect on treatment adjustments should be studied. Trial registration: ClinicalTrials.gov, NCT01041989, retrospectively registered 04/01/2010.

**Supplementary Information:**

The online version contains supplementary material available at 10.1007/s41999-025-01354-1.

## Key summary points

**Aim:**

To study cardiovascular and glucose-lowering medication use, its trajectories along with age, and differences in use between study (lifestyle intervention vs. control), sex, and age groups among older adults at risk of cognitive impairment.

**Findings:**

Cardiovascular and glucose-lowering medication use was common and increased over time. The use was fairly similar between different study and sex groups, but older participants used more cardiovascular medication compared to younger.

**Message:**

The use of cardiovascular and glucose-lowering medication increases along with age among older people at risk of cognitive impairment which reflects the increasing cardiometabolic morbidity, management of the risks and diseases via medication and possible changes in medical practices.

**Supplementary Information:**

The online version contains supplementary material available at 10.1007/s41999-025-01354-1.

## Introduction

Cardiovascular diseases and diabetes are common chronic conditions and contribute remarkably to mortality, health care service utilization and costs worldwide [1–4]. Elevated blood pressure, cholesterol, and glucose levels, along with unhealthy lifestyles, are associated not only with developing cardiovascular and metabolic diseases and events but also with memory disorders such as Alzheimer’s disease [1, 2, 4–6]. Managing these risk factors is therefore important for preventing these chronic diseases and conditions and related deaths [1, 2, 4, 5, 7, 8].

The Finnish Geriatric Intervention Study to Prevent Cognitive Impairment and Disability (FINGER) is a multifactorial lifestyle intervention having shown that by targeting simultaneously several health habits it is possible to enhance cognitive performance [9], reduce the risk of chronic conditions [10] and cardiovascular events [11], maintain mobility and daily functioning [12], and decrease the need for hospital and emergency care [13]. Moreover, lifestyle interventions have been widely shown to be effective in preventing cardiovascular diseases and diabetes [4, 8, 14–17].

In addition to improving lifestyles, the FINGER intervention targeted at the management of cardiovascular and metabolic risk factors through enhanced monitoring and guidance of elevated blood pressure, lipid and glucose levels [18]. When necessary, the participants were advised to contact their health care provider to initiate or adjust medications. Nowadays, there are many effective medications recommended to be used in the treatment of hypertension, dyslipidemia and diabetes [1, 6, 8, 19]. On the other hand, the interventions enhancing healthy lifestyle can reduce the need for medications, especially for antidiabetic medication [20–22]. However, few studies have investigated the effects of lifestyle interventions on cardiovascular medication use in older adults [21, 23], and the effects of FINGER intervention on medication use have not been previously studied.

While cardiovascular and metabolic medications are widely used, studies on medication use among older people and its trajectories along with age are scarce [24, 25]. The aim of this study was to investigate the prevalence of antihypertensive, lipid-lowering, antithrombotic, and glucose-lowering medication use among the FINGER population, i.e. people aged 60–77 years at risk of cognitive impairment, and how the use of these medications developed during a 9-year follow-up. We also studied if the intervention had effects on medication use and if there were differences between sexes and age groups.

## Methods

### FINGER intervention

The FINGER study was a two-year randomized controlled trial conducted in years 2009–2014 (ClinicalTrials.gov NCT01041989) [18]. Participants (*n* = 1259) were randomly assigned into the intervention (*n* = 628) and control (*n* = 631) groups. Study nurses conducted computer-generated randomization at each study site (six cities and their surrounding areas in Finland) after baseline assessment. The inclusion criteria for the study were age of 60–77 years at the start of the study, Cardiovascular Risk Factors, Aging, and Incidence of Dementia (CAIDE) Risk Score of 6 points or higher indicating presence of some modifiable risk factors [26], and cognitive performance at the mean level or slightly lower than expected for age according to Finnish Population norms tested with the Consortium to Establish a Registry for Alzheimer’s Disease neuropsychological battery. Exclusion criteria were previously diagnosed or suspected dementia and disorders affecting safe engagement in the intervention (e.g. malignant disease, major depression, severe loss of vision or hearing, or symptomatic cardiovascular disease) and coincident participation in another intervention trial. The more detailed description of the FINGER study protocol, recruitment, characteristics of the participants, and the results on primary outcomes have been previously reported [9, 18, 27].

Participants in both groups visited study nurse at baseline, 6, 12 and 24 months, and study physician at screening and 24 months for data collection, including assessment of vascular risk factors. Both groups also received a brief intervention on healthy lifestyle at baseline, and general feedback about their laboratory results by mail after the study nurse visits. The multidomain lifestyle intervention included simultaneous nutritional counselling, physical activity, cognitive training, social activities, and cardiovascular risk monitoring and management. These have been described in detail previously [9, 18, 27]. Monitoring and management of metabolic and vascular risk factors were based on national guidelines and aimed to improve blood pressure, lipids, blood glucose, and body weight by improving lifestyles and providing advice related to medications. Participants in the intervention group visited the study nurses (at 3, 9, 18 months) and physicians (at 3, 6, 12 months) who evaluated anthropometric measures, laboratory tests, and cardiovascular and metabolic conditions. The study nurses and physicians also gave advice for the management of cardiovascular and metabolic risks and recommended contacting local healthcare if medication was needed or had to be adjusted. The study physicians did not prescribe medication.

The active intervention period lasted for two years for each participant (during the years 2009–2014) and was followed by a light maintenance intervention during 2016–2018 with weekly text messages on healthy lifestyle tips sent to the intervention participants. Follow-up examinations for both groups were conducted approximately at 5 (during 2015–2016) and at 7 (during 2017–2018) years after the baseline.

### Ethical considerations

The FINGER study was approved by the coordinating ethics committee of the Hospital District of Helsinki and Uusimaa (HUS/1204/2017). The participants gave written informed consent before enrolment in the study including also consent for linking national register data to the clinical trial data.

### Outcome measures

The primary outcome of the FINGER intervention study was cognitive performance, assessed using an extended version of the neuropsychological test battery. The results of the main outcome have been reported previously [9, 18].

In the current study, the focus was on the medication use of FINGER participants. Data were retrieved from the Finnish Social Insurance Institution’s national Prescription Register [28], which includes records of reimbursed medications dispensed from all community pharmacies in Finland since 1994. Over-the-counter medications, non-reimbursed prescription medications, and medications used in hospitals or public nursing homes are not included in the register.

The available data included the dates of medication purchases and Anatomic Therapeutic Chemical (ATC) classification codes of the purchased medications. In Finland, reimbursed medications can be purchased for a maximum of three months at a time. Medication data were analyzed annually starting from the study baseline until the end of the year 2019 or death. Only full follow-up years per participant were included in the analysis. To be defined as a medication user, participants had to have at least two reimbursed purchases within the previous 365 days. The index date was defined as the baseline visit date.

Medications were categorized using ATC codes. Medications examined in this study were cardiovascular medications including antihypertensive medications (ATC codes starting with C02, C03, C07, C08, C09; analyzed both separately and altogether), lipid-lowering medications (statins: ATC codes starting with C10AA and C10B; all lipid-lowering medication: ATC codes starting with C10), and antithrombotic medications (ATC codes starting with B01A). Glucose-lowering medications used to treat diabetes (ATC codes starting with A10A and A10B separately and altogether) were also studied.

Background characteristics were collected through a questionnaire filled by the participants. Physicians interviewed the participants about self-reported diseases at the baseline study visit.

### Statistical analyses

To summarize participants’ characteristics and medication data, descriptive statistics (frequencies, means and standard deviations (SD)) were used. Comparison between participant groups at baseline were conducted using independent-samples t-test for continuous variables and Chi-Square tests for categorical variables.

Generalized Estimating Equations (GEE) using binomial logit link function and unstructured covariance matrix for random effects of baseline and follow-up timepoints (one common effect for 1–9 years) were used to determine if there were any differences in medication use over time, or between different participant groups (intervention vs. control; men vs. women; < 70 years of age vs. ≥ 70 years of age). Analyses were adjusted for age in years at baseline, sex, study group, education in years, and study site. We estimated linear combinations of marginal estimates to compare both changes over time within groups with respect to baseline, and differences between the changes in groups. We present confidence intervals with 95% and *p*-values of these estimates. *p*-values < 0.05 were considered as statistically significant. For sensitivity analyses, we also applied three-way interaction (time, study group, and age group or sex) analyses, but there were no three-way interactions and thus we present only the main results. SPSS version 29.0 and Stata version 18.0 were used in the analyses.

## Results

### Participant characteristics

The mean age of the participants at baseline (*n* = 1259) was 69 years and 53% of them were men (Table 1). Their mean length of education was 10 years. At baseline, two-thirds of the participants were diagnosed with elevated blood pressure (66%) or cholesterol level (67%). Of the participants, 13% were diagnosed with or treated for diabetes, 6% for angina pectoris, 3% for cardiac insufficiency, and 2% for cerebrovascular disease. There were no differences between the intervention and control groups in the background characteristics or disease prevalences at the baseline.

**Table 1:** Participant characteristics at baseline according to intervention allocation

	All (*n* = 1259)	Intervention (*n* = 628)	Control (*n* = 631)	*p*-value^a^
Baseline age (years), mean (SD)	68.8 (4.7)	69.0 (4.7)	68.7 (4.7)	0.261
Men, n (%)	672 (53.4)	345 (54.7)	327 (52.1)	0.354
Education (years), mean (SD)	10.0 (3.5)	10.0 (3.5)	10.0 (3.4)	0.922
Elevated blood pressure (diagnosed at any time earlier), n (%)	831 (66.4)	424 (67.4)	407 (65.4)	0.460
Elevated blood cholesterol level (diagnosed at any time earlier), n (%)	839 (67.2)	410 (65.4)	429 (69.0)	0.178
Myocardial infarction (diagnosed at any time earlier), n (%)	64 (5.1)	33 (5.2)	31 (5.0)	0.823
Stroke, cerebral hemorrhage or obstruction of a cerebral vessel (diagnosed at any time earlier), n (%)	68 (5.4)	33 (5.3)	35 (5.6)	0.782
Cardiac insufficiency (diagnosed or treated during the last 12 months), n (%)	35 (2.8)	18 (2.9)	17 (2.7)	0.887
Effort angina (Angina pectoris) (diagnosed or treated during the last 12 months), n (%)	77 (6.2)	44 (7.0)	33 (5.3)	0.211
Cerebrovascular disease (diagnosed or treated during the last 12 months), n (%)	22 (1.8)	10 (1.6)	12 (1.9)	0.648
Diabetes (diagnosed or treated during the last 12 months), n (%)	168 (13.4)	87 (13.8)	81 (13.0)	0.659

^a^Chi-Square test for categorical variables, independent-samples *t*-test for continuous variables

The average follow-up time per participant from the study baseline visit to the end of year 2019 was 9.3 years. During the follow-up, 157 participants died. Of them, 109 (69%) were men, 88 (56%) were allocated into the control group, and 94 (60%) were 70 years or older at baseline.

### Medication use over time

From baseline to ninth year, the proportion of participants using any cardiovascular or glucose-lowering medication increased from 68 to 81% (*p* < 0.001) (Table 2). At baseline, over half of the participants (56%) used some antihypertensive medication, and the prevalence increased linearly over nine years to 72% (*p* < 0.001 compared to baseline). The most commonly used antihypertensive medications were those acting on the renin-angiotensin system (from 37% at baseline to 51% after nine years, *p* < 0.001) and beta blockers (from 29% at baseline to 40% after nine years, *p* < 0.001). The use of calcium channel blockers increased during the follow-up from 19 to 27% (*p* < 0.001). Use of diuretics remained stable during the first six years (around 10%) but increased to 17% by the ninth year (*p* < 0.001 compared to baseline).

**Table 2:** Prevalences of medication use annually starting from baseline up to nine years (during the years 2009–2019)^a^, and mean age of participants in each study point

	Baseline	1st year	2nd year	3rd year	4th year	5th year	6th year	7th year	8th year	9th year
Number of participants	1259	1256	1246	1234	1223	1204	1183	1162	1133	775
Mean age of participants (SD)	68.8 (4.6)	69.8 (4.6)	70.8 (4.6)	71.8 (4.6)	72.8 (4.6)	73.7 (4.7)	74.7 (4.6)	75.7 (4.6)	76.7 (4.6)	77.3 (4.5)
All studied medications, % (n)	67.8 (854)	69.9 (878)**	70.9 (884)***	71.8 (886)***	73.1 (894)***	74.3 (895)***	75.9 (898)***	77.7 (903)***	79.9 (905)***	80.6 (625)***
Antihypertensive medications, all, % (n)	56.2 (707)	58.7 (737)***	60.0 (748)***	61.3 (756)***	62.1 (759)***	64.8 (780)***	66.2 (783)***	68.8 (800)***	70.7 (801)***	71.5 (554)***
Antihypertensives (C02)^b^, % (n)	0.7 (9)	0.6 (7)	0.7 (9)	0.7 (9)	1.1 (13)	0.8 (10)	0.8 (9)	0.9 (10)	0.8 (9)	0.8 (6)
Diuretics (C03), % (n)	9.5 (119)	9.9 (124)	10.2 (127)	9.4 (116)	8.8 (108)	10.4 (125)	10.8 (128)	12.7 (148)***	13.5 (153)***	16.5 (128)***
Beta blockers (C07), % (n)	28.7 (361)	29.7 (373)	30.7 (383)**	32.3 (398)***	34.1 (417)***	36.2 (436)***	37.1 (439)***	38.2 (444)***	38.3 (434)***	39.5 (306)***
Calcium channel blockers (C08), % (n)	19.1 (240)	19.8 (249)	20.1 (251)	22.0 (271)***	22.6 (277)***	23.3 (281)***	22.9 (271)***	24.2 (281)***	25.3 (287)***	27.1 (210)***
Renin-angiotensin system medications (C09), % (n)	37.1 (467)	39.8 (500)***	41.2 (513)***	42.5 (524)***	42.7 (522)***	44.9 (541)***	46.4 (549)***	49.2 (572)***	50.6 (573)***	51.2 (397)***
Lipid-lowering medications (C10), % (n)	40.7 (512)	41.4 (520)	40.9 (510)	42.9 (530)*	41.8 (511)	42.4 (511)	43.2 (511)*	44.8 (521)**	46.9 (531)***	47.5 (368)***
Statins (C10AA, C10B), % (n)	40.2 (506)	40.8 (513)	40.2 (501)	42.2 (521)*	41.1 (503)	42.0 (506)	42.8 (506)*	44.4 (516)**	45.9 (520)***	46.7 (362)***
Antithrombotic medications (B01), % (n)	6.8 (85)	10.7 (134)***	12.5 (156)***	13.5 (166)***	16.1 (197)***	17.6 (212)***	19.4 (230)***	21.1 (245)***	23.5 (266)***	25.2 (195)***
Glucose-lowering medications, all, % (n)	12.9 (163)	14.2 (178)**	15.1 (188)***	16.3 (201)***	16.8 (205)***	17.3 (208)***	17.7 (209)***	18.1 (210)***	19.6 (222)***	19.2 (149)***
Insulins (A10A)^b^, % (n)	2.8 (35)	3.1 (39)	3.0 (38)	3.0 (37)	3.6 (44)	3.7 (45)	4.1 (48)	4.1 (48)	4.4 (50)	3.9 (30)
Other than insulins (A10B), % (n)	11.9 (150)	13.1 (164)**	14.0 (174)***	15.2 (188)***	15.5 (190)***	16.2 (195)***	16.5 (195)***	17.0 (198)***	18.6 (211)***	18.7 (145)***

^a^GEE used to analyze the difference from baseline (adjusted by study group, age, sex, study site, education)

^b^GEE analyses not conducted due to small number of users

^*^p-value < 0.05, **p-value < 0.01, ***p-value < 0.001

At baseline, 41% of the participants used some lipid-lowering medication (Table 2). The use remained fairly stable for the first six years and then increased to 48% by the end of the follow-up (*p* < 0.001 compared to baseline). Most of these medications were statins as the prevalence of statin use at 40% at baseline and 47% (*p* < 0.001) after nine years.

A small proportion of the participants (7%) used antithrombotic medications at baseline (Table 2). Their use increased linearly over time, reaching 25% after nine years (*p* < 0.001).

Glucose-lowering medication was used by 13% of the participants at baseline (Table 2). The prevalence of glucose-lowering medication excluding insulins was 12%, and the prevalence of insulin use was 3% at baseline. The use of glucose-lowering medication excluding insulins increased over time, reaching 19% after nine years, (*p* < 0.001 compared to baseline).

Discontinuing medication use was not very common. Of those who used antihypertensives at baseline and remained in the study after eight years, 4% (25/617) no longer used the medication during the eighth year. The corresponding percentages were 18% (84/457) for lipid-lowering medication, 18% (12/67) for antithrombotics, and 10% (14/140) for glucose-lowering medication.

### Group differences in medication use

**Fig. 1:**
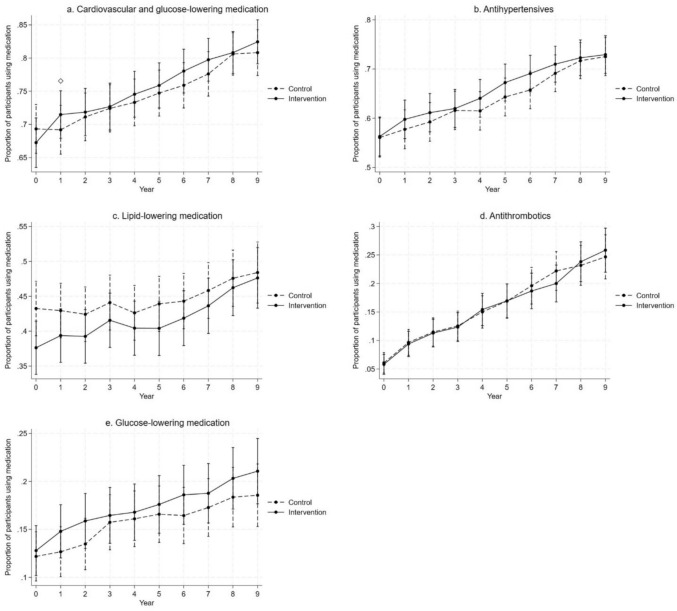
Predicted proportions of participants using any **a** cardiovascular or glucose-lowering, **b** antihypertensive, **c** lipid-lowering, **d** antithrombotic, and **e** glucose-lowering medication during the 9-year follow-up in the intervention and control groups (with 95% CI; adjusted for age, sex, study site, and education). Diamond refers to statistically significant difference in changes from baseline between the groups

During the first year of the intervention, the total use of cardiovascular and glucose-lowering medication increased more in the intervention group compared to the control group (Fig. 1, Online Resource 1). After the first year, trends in use were similar in both groups. There were no statistically significant differences between the intervention and control groups when studying medication groups separately (antihypertensive, lipid-lowering, antithrombotic, and glucose-lowering medications) (Fig. 1, Supplementary Table 1).

**Fig. 2:**
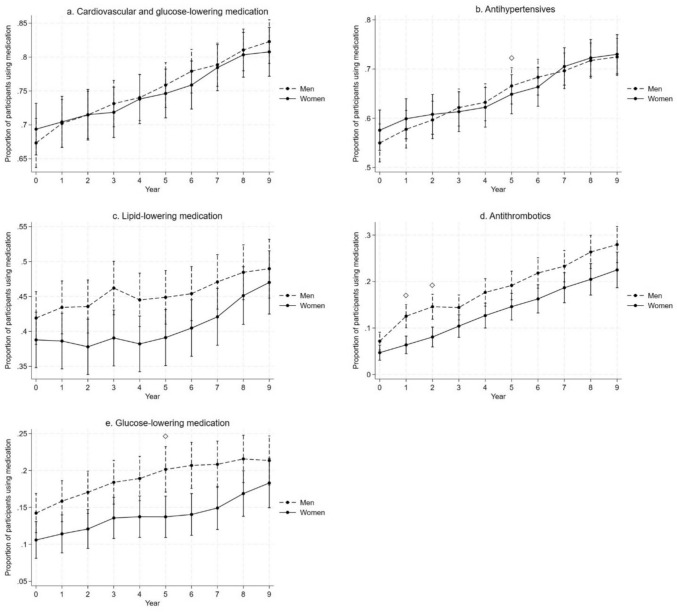
Predicted proportions of participants using any **a** cardiovascular or glucose-lowering, **b** antihypertensive, **c** lipid-lowering, **d** antithrombotic, and **e** glucose-lowering medication during the 9-year follow-up in women and men (with 95% CI; adjusted for age, sex, study site, and education). Diamond refers to statistically significant difference in changes from baseline between the groups

The total use of cardiovascular and glucose-lowering medication was similar between men and women (Fig. 2, Online Resource 2). The use of antihypertensive and glucose-lowering medication increased more among men than among women from baseline to the fifth year and men used glucose-lowering medication more than women in the fifth and sixth years, but not thereafter. Antithrombotic use increased more among men during the first two years and was also more prevalent among men during those years compared to women. There were no longer differences between men and women in the third year, and trends were similar thereafter. There were no differences in prevalence or trends of using lipid-lowering medication between men and women.

**Fig. 3:**
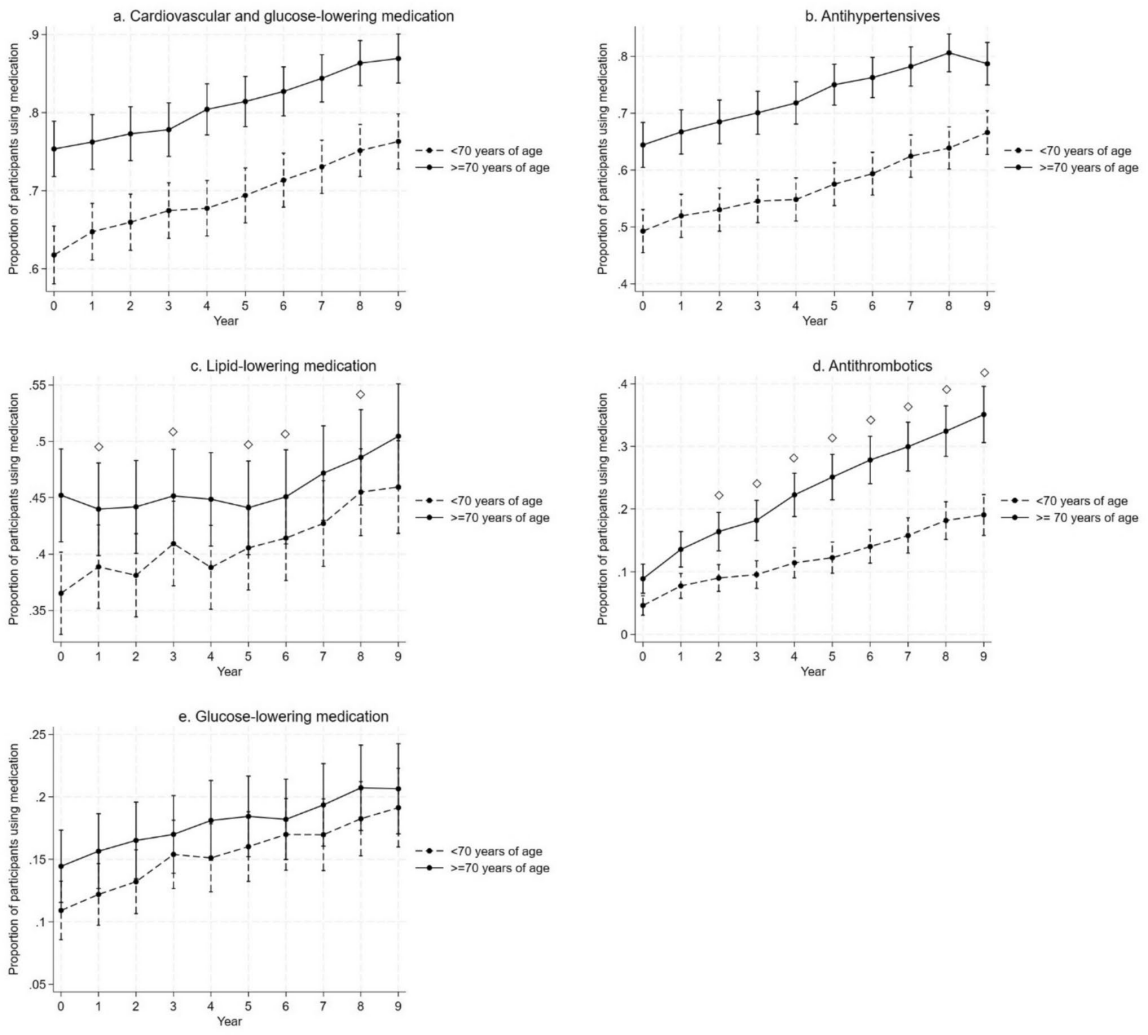
Predicted proportions of participants using any **a** cardiovascular or glucose-lowering, **b** antihypertensive, **c** lipid-lowering, **d** antithrombotic, and **e** glucose-lowering medication during the 9-year follow-up in different age groups (with 95% CI; adjusted for age, sex, study site, and education). Diamond refers to statistically significant difference in changes from baseline between the groups

The total use of cardiovascular and glucose-lowering medication was higher among the older participants (≥ 70 years of age) compared to the younger (< 70 years of age) throughout the follow-up, but increasing trends were similar in both groups (Fig. 3, Online Resource 3). Older participants used more antihypertensive medication than the younger, with similar increasing trends in use. Older participants used lipid-lowering medication more at baseline, but the difference disappeared as use increased among the younger participants and remained stable among the older participants up to the sixth year. Increasing trends in use were similar thereafter. Antithrombotic use was more prevalent among the older participants throughout the follow-up. The use increased in both groups over time, but the increase was greater among the older participants. There were no differences between the age groups in glucose-lowering medication use.

## Discussion

This study provides an overview of the cardiovascular and glucose-lowering medication use during a 9-year follow-up among older people who were at risk of cognitive impairment at the study baseline. According to the results, the use of medications to prevent and treat cardiovascular and metabolic risks and diseases, which means a combination of primary and secondary prevention, was common in the study population. At the beginning of the follow-up, 68% of the participants used some cardiovascular or glucose-lowering medication and after nine years, this proportion was 81%.

Cardiovascular medications are among the most used medications in many countries [29–31]. In line with our results, in Denmark, 62% of older population used cardiovascular medication in 2015 [32]. In Australian older population (65 +), 77% used at least one cardiovascular medication in 2019 [33]. Instead, in China, only 32% of older population used cardiovascular medication in 2018, even though two-thirds of the population had hypertension [34]. Differences in the prevalences of medication use between countries can partly be explained by disease prevalences and national guidelines for treating cardiovascular and metabolic risks and diseases [29, 35, 36]. Also, the pharmaceutical market, availability of medicines, and the population’s economic situation may vary, affecting medication use between countries.

The pharmaceutical market and differences in morbidity and national guidelines also have effects on which cardiovascular medications are most commonly used. In our study, the most used cardiovascular medications were antihypertensives (56% at baseline) and lipid-lowering medication (41%). Among antihypertensives, the use of renin-angiotensin system modifying medications, which are the first-line treatment also according to the national guidelines, was the most prevalent (37%), while diuretics were used the least (10%). In the Danish older population, 33% used statins and 20% used calcium channel blockers in 2015 [32], whereas among Australian older people, 41% used lipid modifiers, 31% used renin-angiotensin system medication, 30% used beta blockers, 24% used diuretics, and 19% used calcium channel blockers in 2019 [33]. In the USA, the most used cardiovascular medications among older adults were lipid-lowering drugs (45%), beta blockers (22%), and angiotensin-converting enzyme inhibitors (21%) in 2015–2016 [31]. Quite similarly, in Canada, the prevalence of lipid-lowering medication use was 34% and of angiotensin-converting enzyme inhibitors 16% in 2016–2017 [31].

Our study showed that the use of cardiovascular and glucose-lowering medication (except for lipid-lowering medication) increases linearly over time among older people at risk of cognitive impairment. This result is in line with previous studies showing that medication use increases with age [24, 25, 32, 37–39]. Ageing itself increases cardiovascular risk, and most people aged 65 and over are at high risk of cardiovascular diseases [1]. However, age is rarely the sole reason for initiating risk-lowering treatments; in fact, target values for blood pressure and glucose level are more lenient for older people. Medication is related to morbidity, so the increased use of preventive and treating medication over time likely reflects increases in risk levels or disease prevalence [25]. Changes in treatment guidelines or medical practices, due to both temporal changes and increased attention to the ageing population, and new pharmacological possibilities may also increase or decrease medication use.

In our study, lipid-lowering medication was the only medication group without increase throughout the follow-up, but its use started to increase after six years. The use of statins, the most commonly used lipid-lowering medications, among older people is under discussion, and there are conflicting perceptions for the use of lipid-lowering medication among older people [1, 40]. However, recent suggestions indicate that there is no reason to discontinue statin treatment in older people if there are no adverse effects significantly impacting their quality of life [40]. In addition, initiating statins as secondary prevention is reasonable even for patients over 75 years. Previous studies have shown contradictory results regarding lipid-lowering medication use along with age. In a study comparing four European countries, lipid-lowering medication use increased between the ages of 65–74 and 75–84 but slightly decreased in the 85 + age group in all countries [29]. Among adults with type 2 diabetes in Germany, use of lipid-lowering medication increased the most among cardiovascular medications in the oldest age group (aged 65 years and over) [25]. Among older people aged 75–90 years in Sweden, lipid-lowering medication use decreased with age [30]. Interestingly, in our study, the use of lipid-lowering medication started to increase during the latter part of the follow-up, despite almost a fifth of the users at baseline discontinuing medication use. In 2016, the European Society of Cardiology (ESC) lowered the target level for blood cholesterol, which also reduced the threshold for prescribing lipid-lowering medications [1]. Finnish national guidelines have closely followed the ESC one, and this may have contributed to the increased use observed in our study. A Finnish study found that statin discontinuation was associated with higher age and female gender, whereas concomitant cardiovascular medication use was associated with a decreased risk of discontinuation [41]. In our study, over 70% of participants used antihypertensives at the end of the follow-up, which may also explain the increasing use of lipid-lowering medication seen in the study.

According to our results, older participants used more cardiovascular and glucose-lowering medications throughout the follow-up compared to younger participants, but the increasing trends were rather similar in both age groups. The exception was lipid-lowering medication whose use increased more in the younger group and the use of antithrombotic medication, which increased more in the older group. Our findings on increasing medication use are consistent with some other studies [29–32, 42] suggesting, together with relatively low discontinuation rates, that deprescribing is not very common among older people, even though blood pressure, body weight, and lipid levels generally decrease along with age and also before possible cognitive decline [5].

In our study, the multidomain lifestyle intervention had an increasing effect on the total use of cardiovascular and glucose-lowering medication during the first year of the trial, but after that, there were no statistically significant differences in medication use trends between the intervention and control groups. The FINGER intervention was effective in preventing cerebrovascular events over seven years in the whole study population, and also total cardiovascular events among those with a history of cardiovascular diseases at baseline [11]. It has also been shown that the intervention enhanced healthy lifestyle during the intervention and also during the 10-year follow-up [43]. However, our study found no differences in cardiovascular medication use between the intervention and control groups during the 9-year follow-up. In a study from USA focusing on men aged 40–75 years of age, healthier lifestyle prevented coronary events regardless of use of medication for coronary risk factors [44]. Our results support this conclusion. However, healthy lifestyle combined with medication use may be more effective in reducing cardiovascular disease risk than either approach alone [44]. When interpreting our results regarding the intervention effect, it must be taken into account that only differences in prevalences of medication use at the ATC group levels were studied. Combination treatment is highly recommended particularly when treating high blood pressure [1], so this study lacks information on potential effects of the intervention on treatment adjustments (e.g. increases or decreases in the number of medications used or in dosing). Furthermore, the intervention may have had varying effects on medication use; improved lifestyle may have reduced the need for medication among some participants while enhanced monitoring and management of cardiovascular and metabolic risk factors may have increased it among others.

Our findings on the intervention effect are partly inconsistent with a few other studies focused on the effect of lifestyle interventions on medication use, which may be partly explained by differences in study populations. Our study focused on older people with some cardiometabolic risks. In a German non-randomized controlled trial aimed at improving lifestyles in the general population, the intervention had a decreasing effect on antihypertensive medication use and no effects on the use of diabetes or lipid-lowering medication [23]. However, that study had a small sample size and low medication use. The Look AHEAD randomized controlled trial, which targeted overweight or obese adults with type 2 diabetes, found that a lifestyle intervention significantly reduced the use of cardiovascular and diabetes medication in a 10-year follow-up, but the study group was younger [21] compared to our study.

There are known disparities in cardiovascular risk factors and mortality by sex [4]. Men have a higher risk of cardiovascular diseases compared to women, and in high-income countries, they also die more frequently from these conditions. Hypertension is more prevalent among men up to 75 years of age, after which it becomes more common in women [6]. In our study, the overall use of cardiovascular and glucose-lowering medication was fairly similar between men and women, even though the use of glucose-lowering and antithrombotic medication was more prevalent and increased more among men in some years during the follow-up. One possible explanation is the exclusion of older adults with symptomatic cardiovascular diseases from our study. Even though women generally have more disabling chronic diseases and they also use more medications than men, men tend to experience more severe and fatal conditions [24, 31, 45]. Previous studies have shown that cardiovascular medication use among older men and women varies across countries. For example, in England, men used lipid-lowering medication more often, whereas in Poland, Portugal and Slovakia, the opposite was observed [29]. Among German patients with type 2 diabetes, more men used antithrombotics and lipid-lowering medications, while women more frequently used antihypertensive medications [25]. In a cross-sectional study in Greenland, antihypertensive medication use was more common among women than men [42]. These differences in results are partly explained by variations in study populations as some studies investigated the general population while others focused on patients with specific diseases or medications.

## Strengths and limitations

Our study provides unique longitudinal data on medication use among older people at risk of cognitive impairment partly due to cardiometabolic factors. Longitudinal studies on medication use are scarce, and trends are typically assessed using different cross-sectional populations at different time points [24, 46]. In addition, age group differences are studied cross-sectionally rather than longitudinally [30].

Medication use in this study was determined based on reimbursed purchases from a national register. However, we had limited data from the register (e.g. no information on the quantity of medication purchased at a time), so we could not estimate the temporal coverage of medication use. Therefore, we defined a user as someone with at least two purchases in a year. This is more reliable than one-purchase-per-year definition used in some studies [29, 32, 38]. A limitation of using register data is that purchases do not necessarily reflect the actual use of medication. This is particularly relevant for cardiovascular medications (e.g. lipid-lowering and antihypertensive medication), where a significant proportion of patients do not adhere to medication use [1]. In addition, not all prescription medications are recorded in the Prescription Register. For example, low-dose acetylsalicylic acid is not reimbursed in Finland and is therefore not recorded, except for combination products with dipyridamole. Changes in reimbursement status over time may also affect how medication purchases are recorded during the follow-up. Nevertheless, most cardiovascular and glucose-lowering medications are reimbursed in Finland, so our study offers a comprehensive overview of their use over time. In addition, using register data eliminates recall bias. We analyzed medication groups rather than individual medications, which provides an overall understanding of the medication use and allows for switches within each group.

There may be selection bias in the study population, as participants in intervention studies tend to be healthier and more educated, potentially leading to differences in disease and medication prevalences compared to the general Finnish older population. However, the FINGER trial’s inclusion criteria favored less educated individuals with higher cardiovascular risk, which may offset this bias. There is no comprehensive data on cardiovascular disease or medication use prevalence among older people in Finland, making it difficult to assess the generalizability of our findings. According to the Finnish Health 2011 study, the prevalence of elevated blood pressure, dyslipidemia, self-reported angina pectoris or heart attack, heart insufficiency, diabetes, and stroke among those aged 65–74 years were 68%, 69%, 21%, 4%, 20%, and 7% for men, and 75%, 67%, 7%, 2%, 14%, and 4%, for women, respectively [47]. These figures suggest that our participants were somewhat healthier in terms of cardiovascular diseases and diabetes, although hypertension and dyslipidemia rates were similar. The exclusion of individuals with symptomatic cardiovascular disease in the FINGER trial may have led to an underestimation of cardiovascular medication use compared to the general Finnish older population. However, the inclusion criteria (increased cardiometabolic risk) may have balanced this underestimation. Additionally, dropouts due to death may have led to lower prevalence estimates in later years, as sicker individuals tend to die earlier and also use more medications.

## Conclusions

Cardiovascular and glucose-lowering medication use increases along with age in the population at risk of cognitive impairment, reflecting both the increase in cardiovascular and metabolic morbidity and, potentially, improvements in risk and disease management. Changes in medical practices over time or in the treatment of ageing population may also contribute to the increase. The FINGER intervention had no effects on the prevalences of medication use, but future studies should investigate the effects of the lifestyle intervention on medication adjustments. In this study, only cardiovascular and glucose-lowering medications were investigated. In the future, the effect of the FINGER intervention on total medication use should be studied.

## Supplementary Information

Below is the link to the electronic supplementary material.

Supplementary file1 (PDF 97 KB)

Supplementary file2 (PDF 91 KB)

Supplementary file3 (PDF 91 KB)

## Data Availability

The datasets presented in this article are not readily available because Public deposition of the de-identified data set is not possible due to legal and ethical reasons, and complete deidentification is not possible as this investigation is part of an ongoing study. The study participants gave informed consent which includes data use only under confidentiality agreement. Further, the data contains large amount of sensitive information and public data deposition may pose privacy concerns. Those fulfilling the requirements for viewing confidential data as required by the Finnish law and the Finnish Institute for Health and Welfare are able to access the data after completion of material transfer agreement. Requests to access the datasets should be directed to kirjaamo@thl.fi.
