# Correction: Cardiovascular and glucose-lowering medication use among older adults: results from 9-year follow-up of the FINGER trial

**DOI:** 10.1007/s41999-025-01397-4

**Published:** 2025-12-24

**Authors:** Maria Sääskilahti, Emma Aarnio, Esko Levälahti, Jenni Lehtisalo, Miia Kivipelto, Timo Strandberg, Riitta Antikainen, Hilkka Soininen, Tiina Laatikainen, Jaakko Tuomilehto, Alina Solomon, Francesca Mangialasche, Tiia Ngandu

**Affiliations:** 1 Department of Public Health, Lifestyles and Living Environments, Finnish Institute for Health and Welfare, 00271 Helsinki, Finland; 2 School of Pharmacy, University of Eastern Finland, 70211 Kuopio, Finland; 3 Institute of Public Health and Clinical Nutrition, University of Eastern Finland, Kuopio, Finland; 4 Division of Clinical Geriatrics, Center for Alzheimer Research, Department of Neurobiology, Care Sciences and Society, Karolinska Institutet, 171 64 Solna, Stockholm, Sweden; 5 The Ageing Epidemiology Research Unit, School of Public Health, Imperial College London, London, UK; 6 University of Helsinki and Helsinki University Hospital, Helsinki, Finland; 7 Research Unit of Population Health/Geriatrics, University of Oulu, 90014 Oulu, Finland; 8 Medical Research Center Oulu, Oulu University Hospital, Oulu, Finland; 9 Department of Clinical Medicine/Neurology, University of Eastern Finland, 70211 Kuopio, Finland; 10 Department of Neurology, Kuopio University Hospital, Kuopio, Finland; 11 Wellbeing Services County of North Karelia (Siun Sote), Joensuu, Finland; 12 South Ostrobothnia Central Hospital, Seinäjoki, Finland; 13 Department of Public Health, University of Helsinki, Helsinki, Finland; 14 Theme Inflammation and Aging, Medical Unit Aging, Karolinska University Hospital, Stockholm, Sweden

**Correction: European Geriatric Medicine** 10.1007/s41999-025-01354-1

In the original version of this article, the given and family names of all authors were incorrectly structured:

Sääskilahti Maria1 · Aarnio Emma2 · Levälahti Esko1 · Lehtisalo Jenni1,3 · Kivipelto Miia4,5 · Strandberg Timo6,7 · Antikainen Riitta7,8 · Soininen Hilkka9,10 · Laatikainen Tiina3,11 · Tuomilehto Jaakko12,13 · Solomon Alina4,9 · Mangialasche Francesca4,14 · Ngandu Tiia1,3

The correct structured author names are listed here:

Maria Sääskilahti1 · Emma Aarnio2 · Esko Levälahti1 · Jenni Lehtisalo1,3 · Miia Kivipelto4,5 · Timo Strandberg6,7 · Riitta Antikainen7,8 · Hilkka Soininen9,10 · Tiina Laatikainen3,11 · Jaakko Tuomilehto12,13 · Alina Solomon4,9 · Francesca Mangialasche4,14 · Tiia Ngandu1,3

The original article has been corrected.

