# Supplementary material for: Cardiovascular and glucose-lowering medication use among older adults: results from 9-year follow-up of the FINGER trial
Source: Eur Geriatr Med. 2025 Dec 3;17(2):961–73. doi: 10.1007/s41999-025-01354-1 (PMC13109183; doi:10.1007/s41999-025-01354-1)
Supplement: Supplementary file 1 — Supplementary file1 (PDF 97 KB) [file 41999_2025_1354_MOESM1_ESM.pdf]

Online resource 1

European Geriatric Medicine

Cardiovascular and glucose-lowering medication use among older adults: results from 9-year follow-up of the FINGER trial

Sääskilahti Maria[1], Aarnio Emma, Levälahti Esko, Lehtisalo Jenni, Kivipelto Miia, Strandberg Timo, Antikainen Riitta, Soininen Hilkka, Laatikainen Tiina, Tuomilehto Jaakko, Solomon Alina, Mangialasche Francesca, Ngandu Tiia

[1]Corresponding author: Sääskilahti Maria, Department of Public Health, Lifestyles and Living Environments, Finnish Institute for Health and Welfare, Helsinki, 00271, Finland, maria.saaskilahti@thl.fi

Table 1. Temporal changes in proportions of participants using medication in intervention and control groups, and differences between the changes in groups during the 9-year follow-up. Adjusted by age, sex, study site, and education.

	Intervention		Control		Difference between groups (intervention-control)	
	Predicted change (95% CI)	p-value	Predicted change (95% CI)	p-value	Predicted difference (95% CI)	p-value
All studied medication						
1y-baseline	0.042 (0.023, 0.062)	<0.001	-0.001 (-0.021, 0.018)	0.893	0.044 (0.016, 0.072)	0.002
2y-baseline	0.046 (0.022, 0.070)	<0.001	0.018 (-0.005, 0.042)	0.132	0.028 (-0.005, 0.061)	0.101
3y-baseline	0.054 (0.028, 0.080)	<0.001	0.031 (0.005, 0.057)	0.019	0.023 (-0.013, 0.060)	0.211
4y-baseline	0.073 (0.046, 0.100)	<0.001	0.040 (0.013, 0.067)	0.004	0.033 (-0.005, 0.072)	0.093
5y-baseline	0.086 (0.058, 0.115)	<0.001	0.054 (0.026, 0.082)	<0.001	0.032 (-0.008, 0.072)	0.117
6y-baseline	0.108 (0.077, 0.138)	<0.001	0.066 (0.035, 0.096)	<0.001	0.042 (-0.001, 0.085)	0.054
7y-baseline	0.125 (0.093, 0.157)	<0.001	0.083 (0.051, 0.115)	<0.001	0.042 (-0.003, 0.087)	0.069
8y-baseline	0.136 (0.101, 0.170)	<0.001	0.113 (0.079, 0.147)	<0.001	0.023 (-0.026, 0.071)	0.356
9y-baseline	0.152 (0.115, 0.189)	<0.001	0.115 (0.078, 0.152)	<0.001	0.037 (-0.015, 0.089)	0.163
Antihypertensives						
1y-baseline	0.035 (0.016, 0.053)	<0.001	0.017 (-0.002, 0.035)	0.080	0.018 (-0.008, 0.044)	0.172
2y-baseline	0.048 (0.025, 0.072)	<0.001	0.032 (0.008, 0.056)	0.009	0.017 (-0.017, 0.050)	0.330
3y-baseline	0.057 (0.031, 0.083)	<0.001	0.055 (0.029, 0.081)	<0.001	0.002 (-0.035, 0.039)	0.922
4y-baseline	0.078 (0.049, 0.106)	<0.001	0.054 (0.025, 0.083)	<0.001	0.023 (-0.017, 0.064)	0.256
5y-baseline	0.110 (0.080, 0.139)	<0.001	0.082 (0.052, 0.112)	<0.050	0.027 (-0.015, 0.069)	0.207
6y-baseline	0.128 (0.096, 0.160)	<0.001	0.097 (0.064, 0.129)	<0.001	0.031(-0.015, 0.077)	0.183
7y-baseline	0.147 (0.114, 0.181)	<0.001	0.130 (0.097, 0.164)	<0.001	0.017 (-0.031, 0.064)	0.494

8y-baseline	0.160 (0.125, 0.195)	<0.001	0.156 (0.121, 0.192)	<0.001	0.004 (-0.046, 0.054)	0.879
9y-baseline	0.166 (0.129, 0.203)	<0.001	0.164 (0.127, 0.202)	<0.001	0.002 (-0.050, 0.054)	0.941
Lipid-lowering medication						
1y-baseline	0.017 (-0.005, 0.040)	0.132	-0.003 (-0.026, 0.020)	0.799	0.020 (-0.012, 0.053)	0.217
2y-baseline	0.016 (-0.011, 0.044)	0.250	-0.008 (-0.037, 0.020)	0.566	0.025 (-0.015, 0.064)	0.224
3y-baseline	0.039 (0.009, 0.069)	0.011	0.008 (-0.022, 0.039)	0.591	0.031 (-0.012, 0.074)	0.161
4y-baseline	0.028 (-0.005, 0.061)	0.092	-0.006 (-0.040, 0.027)	0.713	0.034 (-0.012, 0.081)	0.149
5y-baseline	0.028 (-0.006, 0.061)	0.103	0.007 (-0.027, 0.041)	0.703	0.021 (-0.027, 0.069)	0.386
6y-baseline	0.042 (0.008, 0.077)	0.016	0.011 (-0.025, 0.046)	0.557	0.032 (-0.017, 0.081)	0.204
7y-baseline	0.060 (0.026, 0.094)	0.001	0.026 (-0.009, 0.061)	0.148	0.034 (-0.015, 0.083)	0.170
8y-baseline	0.086 (0.049, 0.123)	0.000	0.043 (0.006, 0.081)	0.024	0.043 (-0.010, 0.095)	0.112
9y-baseline	0.100 (0.059, 0.141)	0.000	0.051 (0.010, 0.093)	0.016	0.048 (-0.010, 0.107)	0.105
Antithrombotics						
1y-baseline	0.036 (0.019, 0.052)	<0.001	0.036 (0.020, 0.052)	<0.001	-0.000 (-0.023, 0.023)	0.989
2y-baseline	0.054 (0.035, 0.073)	<0.001	0.055 (0.036, 0.074)	<0.001	0.001 (-0.026, 0.028)	0.955
3y-baseline	0.065 (0.043, 0.086)	<0.001	0.065 (0.044, 0.086)	<0.001	0.001 (-0.029, 0.031)	0.968
4y-baseline	0.089 (0.065, 0.114)	<0.001	0.096 (0.072, 0.120)	<0.001	0.007 (-0.027, 0.041)	0.696
5y-baseline	0.108 (0.081, 0.136)	<0.001	0.111 (0.085, 0.138)	<0.001	0.003 (-0.035, 0.041)	0.876
6y-baseline	0.135 (0.106, 0.165)	<0.001	0.129 (0.100, 0.157)	<0.001	-0.007 (-0.047, 0.034)	0.750
7y-baseline	0.161 (0.129, 0.193)	<0.001	0.142 (0.112, 0.172)	<0.001	-0.019 (-0.063, 0.024)	0.385
8y-baseline	0.171 (0.138, 0.204)	<0.001	0.180 (0.147, 0.213)	<0.001	0.009 (-0.038, 0.056)	0.706
9y-baseline	0.186 (0.148, 0.223)	<0.001	0.200 (0.163, 0.237)	<0.001	0.014 (-0.038, 0.067)	0.593
Glucose-lowering medication						
1y-baseline	0.020 (0.001, 0.031)	<0.001	0.005 (-0.006, 0.016)	0.380	0.015 (-0.000, 0.031)	0.055
2y-baseline	0.031 (0.017, 0.045)	<0.001	0.013 (-0.001, 0.027)	0.063	0.018 (-0.002, 0.038)	0.074
3y-baseline	0.037 (0.019, 0.055)	<0.001	0.036 (0.018, 0.053)	<0.001	0.001 (-0.024, 0.027)	0.929
4y-baseline	0.040 (0.021, 0.059)	<0.001	0.039 (0.021, 0.058)	<0.001	0.001 (-0.026, 0.027)	0.951
5y-baseline	0.048 (0.028, 0.068)	<0.001	0.044 (0.024, 0.064)	<0.001	0.004 (-0.024, 0.032)	0.772
6y-baseline	0.058 (0.036, 0.080)	<0.001	0.043 (0.022, 0.064)	<0.001	0.016 (-0.015, 0.046)	0.315
7y-baseline	0.060 (0.037, 0.082)	<0.001	0.051 (0.029, 0.073)	<0.001	0.009 (-0.023, 0.041)	0.587
8y-baseline	0.075 (0.050, 0.100)	<0.001	0.062 (0.038, 0.086)	<0.001	0.014 (-0.021, 0.048)	0.442
9y-baseline	0.083 (0.054, 0.111)	<0.001	0.064 (0.036, 0.091)	<0.001	0.019 (-0.021, 0.059)	0.349
