# Supplementary material for: Cardiovascular and glucose-lowering medication use among older adults: results from 9-year follow-up of the FINGER trial
Source: Eur Geriatr Med. 2025 Dec 3;17(2):961–73. doi: 10.1007/s41999-025-01354-1 (PMC13109183; doi:10.1007/s41999-025-01354-1)
Supplement: Supplementary file 2 — Supplementary file2 (PDF 91 KB) [file 41999_2025_1354_MOESM2_ESM.pdf]

Online resource 2

European Geriatric Medicine

Cardiovascular and glucose-lowering medication use among older adults: results from 9-year follow-up of the FINGER trial

Sääskilahti Maria[1], Aarnio Emma, Levälahti Esko, Lehtisalo Jenni, Kivipelto Miia, Strandberg Timo, Antikainen Riitta, Soininen Hilkka, Laatikainen Tiina, Tuomilehto Jaakko, Solomon Alina, Mangialasche Francesca, Ngandu Tiia

[1]Corresponding author: Sääskilahti Maria, Department of Public Health, Lifestyles and Living Environments, Finnish Institute for Health and Welfare, Helsinki, 00271, Finland, maria.saaskilahti@thl.fi

Table 1. Temporal changes in proportions of participants using medication among men and women, and differences between the changes in groups during the 9-year follow-up. Adjusted by age, study group, study site, and education.

	Women		Men		Difference between groups (women-men)	
	Predicted change (95% CI)	p-value	Predicted change (95% CI)	p-value	Predicted difference (95% CI)	p-value
All studied medication						
1y-baseline	0.011 (-0.010, 0.031)	0.304	0.029 (0.010, 0.048)	0.003	-0.018 (-0.046, 0.010)	0.205
2y-baseline	0.021 (-0.003, 0.046)	0.087	0.041 (0.018, 0.064)	<0.001	-0.020 (-0.053, 0.014)	0.243
3y-baseline	0.025 (-0.002, 0.052)	0.069	0.058 (0.033, 0.083)	<0.001	-0.033 (-0.070, 0.004)	0.077
4y-baseline	0.044 (0.016, 0.072)	0.002	0.067 (0.040, 0.093)	<0.001	-0.022 (-0.061, 0.016)	0.256
5y-baseline	0.053 (0.023, 0.082)	<0.001	0.085 (0.058, 0.113)	<0.001	-0.033 (-0.073, 0.008)	0.112
6y-baseline	0.065 (0.034, 0.097)	<0.001	0.106 (0.076, 0.135)	<0.001	-0.041 (-0.084, 0.002)	0.064
7y-baseline	0.091 (0.058, 0.124)	<0.001	0.115 (0.084, 0.146)	<0.001	-0.025 (-0.070, 0.021)	0.289
8y-baseline	0.110 (0.074, 0.145)	<0.001	0.137 (0.104, 0.170)	<0.001	-0.027 (-0.076, 0.021)	0.269
9y-baseline	0.114 (0.076, 0.152)	<0.001	0.149 (0.114, 0.185)	<0.001	-0.035 (-0.088, 0.017)	0.182
Antihypertensives						
1y-baseline	0.023 (0.004, 0.042)	0.017	0.028 (0.010, 0.046)	0.002	-0.004 (0.031, 0.022)	0.740
2y-baseline	0.032 (0.008, 0.057)	0.010	0.047 (0.024, 0.070)	<0.001	-0.015 (-0.048, 0.019)	0.397
3y-baseline	0.038 (0.010, 0.065)	0.007	0.072 (0.046, 0.097)	<0.001	-0.034 (-0.072, 0.003)	0.071
4y-baseline	0.047 (0.017, 0.076)	0.002	0.083 (0.055, 0.110)	<0.001	-0.036 (-0.076, 0.004)	0.081
5y-baseline	0.073 (0.042, 0.104)	<0.001	0.116 (0.087, 0.145)	<0.001	-0.043 (-085.0, -0.000)	0.047
6y-baseline	0.088 (0.054, 0.122)	<0.001	0.134 (0.102, 0.165)	<0.001	-0.046 (-0.092, 0.001)	0.053
7y-baseline	0.130 (0.095, 0.164)	<0.001	0.147 (0.114, 0.179)	<0.001	-0.017 (-064.0, 0.031)	0.483
8y-baseline	0.147 (0.111, 0.183)	<0.001	0.167 (0.133, 0.202)	<0.001	-0.021 (-0.071, 0.029)	0.419

9y-baseline	0.154 (0.116, 0.193)	<0.001	0.175 (0.139, 0.211)	<0.001	-0.020 (-0.073, 0.032)	0.446
Lipid-lowering medication						
1y-baseline	-0.002 (-0.025, 0.022)	0.891	0.015 (-0.007, 0.038)	0.184	-0.017 (-0.049, 0.016)	0.310
2y-baseline	-0.010 (-0.038, 0.019)	0.499	0.017 (-0.011, 0.044)	0.238	-0.026 (-0.066, 0.013)	0.192
3y-baseline	0.003 (-0.028, 0.034)	0.863	0.043 (0.013, 0.073)	0.005	-0.040 (-0.083, 0.003)	0.068
4y-baseline	-0.006 (-0.039, 0.028)	0.739	0.026 (-0.006, 0.058)	0.115	-0.032 (-0.078, 0.015)	0.183
5y-baseline	0.003 (-0.031, 0.038)	0.851	0.030 (-0.003, 0.063)	0.079	-0.026 (-0.074, 0.021)	0.280
6y-baseline	0.017 (-0.018, 0.052)	0.349	0.035 (0.001, 0.069)	0.045	-0.018 (-0.067, 0.031)	0.475
7y-baseline	0.033 (-0.002, 0.068)	0.067	0.052 (0.018, 0.086)	0.003	-0.019 (-0.068, 0.030)	0.454
8y-baseline	0.063 (0.025, 0.101)	0.001	0.065 (0.029, 0.102)	<0.001	-0.002 (-0.055, 0.051)	0.938
9y-baseline	0.082 (0.039, 0.125)	<0.001	0.071 (0.030, 0.111)	0.001	0.012 (-0.047, 0.070)	0.699
Antithrombotics						
1y-baseline	0.017 (0.003, 0.030)	0.017	0.054 (0.035, 0.072)	<0.001	-0.037 (-0.060, 0.015)	0.001
2y-baseline	0.034 (0.017, 0.050)	<0.001	0.074 (0.053, 0.095)	<0.001	-0.040 (-0.067, 0.014)	0.003
3y-baseline	0.057 (0.037, 0.077)	<0.001	0.072 (0.050, 0.095)	<0.001	-0.015 (-0.045, 0.015)	0.320
4y-baseline	0.080 (0.057, 0.103)	<0.001	0.105 (0.079, 0.130)	<0.001	-0.025 (-0.059, 0.009)	0.149
5y-baseline	0.099 (0.073, 0.125)	<0.001	0.120 (0.092, 0.148)	<0.001	-0.021 (-0.059, 0.170)	0.281
6y-baseline	0.116 (0.088, 0.143)	<0.001	0.147 (0.117, 0.177)	<0.001	-0.031 (-0.071, 0.009)	0.133
7y-baseline	0.140 (0.110, 0.170)	<0.001	0.161 (0.129, 0.193)	<0.001	-0.022 (-0.065, 0.022)	0.335
8y-baseline	0.158 (0.126, 0.190)	<0.001	0.192 (0.158, 0.226)	<0.001	-0.034 (-0.081, 0.013)	0.152
9y-baseline	0.178 (0.141, 0.215)	<0.001	0.208 (0.170, 0.246)	<0.001	-0.030 (-0.082, 0.023)	0.269
Glucose-lowering medication						
1y-baseline	0.008 (-0.002, 0.019)	0.126	0.016 (0.005, 0.028)	0.006	-0.008 (-0.023, 0.008)	0.322
2y-baseline	0.015 (0.002, 0.028)	0.028	0.028 (0.014, 0.043)	<0.001	-0.013 (-0.033, 0.006)	0.186
3y-baseline	0.030 (0.013, 0.047)	0.001	0.042 (0.023, 0.060)	<0.001	-0.012 (-0.037, 0.014)	0.359
4y-baseline	0.032 (0.014, 0.049)	0.001	0.047 (0.028, 0.066)	<0.001	-0.015 (-0.042, 0.011)	0.253
5y-baseline	0.031 (0.013, 0.050)	0.001	0.059 (0.039, 0.080)	<0.001	-0.028 (-0.056, -0.000)	0.049
6y-baseline	0.035 (0.014, 0.055)	0.001	0.065 (0.042, 0.087)	<0.001	-0.030 (-0.060, -0.000)	0.050
7y-baseline	0.043 (0.022, 0.065)	<0.001	0.066 (0.043, 0.089)	<0.001	-0.023 (-0.054, 0.009)	0.157
8y-baseline	0.063 (0.039, 0.087)	<0.001	0.073 (0.048, 0.098)	<0.001	-0.010 (-0.044, 0.024)	0.551
9y-baseline	0.077 (0.049, 0.105)	<0.001	0.071 (0.043, 0.100)	<0.001	0.006 (-0.034, 0.046)	0.770
