# Supplementary material for: Cardiovascular and glucose-lowering medication use among older adults: results from 9-year follow-up of the FINGER trial
Source: Eur Geriatr Med. 2025 Dec 3;17(2):961–73. doi: 10.1007/s41999-025-01354-1 (PMC13109183; doi:10.1007/s41999-025-01354-1)
Supplement: Supplementary file 3 — Supplementary file3 (PDF 91 KB) [file 41999_2025_1354_MOESM3_ESM.pdf]

Online resource 3

European Geriatric Medicine

Cardiovascular and glucose-lowering medication use among older adults: results from 9-year follow-up of the FINGER trial

Sääskilahti Maria[1], Aarnio Emma, Levälahti Esko, Lehtisalo Jenni, Kivipelto Miia, Strandberg Timo, Antikainen Riitta, Soininen Hilkka, Laatikainen Tiina, Tuomilehto Jaakko, Solomon Alina, Mangialasche Francesca, Ngandu Tiia

[1]Corresponding author: Sääskilahti Maria, Department of Public Health, Lifestyles and Living Environments, Finnish Institute for Health and Welfare, Helsinki, 00271, Finland, maria.saaskilahti@thl.fi

Table 1. Temporal changes in proportions of participants using medication in older (≥70 years of age) and younger (<70 years of age) age groups, and differences between the changes in groups during the 9-year follow-up. Adjusted by sex, study group, study site, and education.

	≥70-year-old		<70-year-old		Difference between groups (older-younger)	
	Predicted change (95% CI)	p-value	Predicted change (95% CI)	p-value	Predicted difference (95% CI)	p-value
All studied medication						
1y-baseline	0.009 (-0.011, 0.029)	0.373	0.030 (0.018, 0.050)	0.004	-0.021 (-0.049, 0.007)	0.148
2y-baseline	0.019 (-0.003, 0.042)	0.096	0.042 (0.018, 0.066)	0.001	-0.023 (-0.056, 0.011)	0.182
3y-baseline	0.025 (-0.000, 0.050)	0.051	0.057 (0.031, 0.083)	<0.001	-0.032 (-0.068, 0.003)	0.078
4y-baseline	0.051 (0.025, 0.076)	<0.001	0.060 (0.033, 0.087)	<0.001	-0.009 (-0.046, 0.028)	0.630
5y-baseline	0.061 (0.034, 0.088)	<0.001	0.076 (0.048, 0.105)	<0.001	-0.016 (-0.055, 0.024)	0.438
6y-baseline	0.074 (0.045, 0.102)	<0.001	0.096 (0.065, 0.126)	<0.001	-0.022 (-0.064, 0.020)	0.299
7y-baseline	0.090 (0.060, 0.120)	<0.001	0.113 (0.081, 0.145)	<0.001	-0.023 (-0.066, 0.021)	0.314
8y-baseline	0.110 (0.078, 0.142)	<0.001	0.134 (0.099, 0.168)	<0.001	-0.024 (-0.071, 0.023)	0.322
9y-baseline	0.116 (0.081, 0.151)	<0.001	0.145 (0.108, 0.183)	<0.001	-0.030 (-0.081, 0.022)	0.258
Antihypertensives						
1y-baseline	0.023 (0.004, 0.042)	0.019	0.027 (0.008, 0.045)	0.005	-0.004 (-0.031, 0.023)	0.773
2y-baseline	0.041 (0.017, 0.065)	0.001	0.038 (0.014, 0.061)	0.002	0.003 (-0.030, 0.036)	0.863
3y-baseline	0.057 (0.031, 0.082)	<0.001	0.053 (0.027, 0.078)	<0.001	0.004 (-0.032, 0.040)	0.836
4y-baseline	0.074 (0.046, 0.102)	<0.001	0.056 (0.028, 0.083)	<0.001	0.018 (-0.021, 0.058)	0.358
5y-baseline	0.106 (0.077, 0.135)	<0.001	0.083 (0.054, 0.111)	<0.001	0.023 (-0.018, 0.064)	0.263
6y-baseline	0.119 (0.087, 0.150)	<0.001	0.101 (0.069, 0.133)	<0.001	0.018 (-0.027, 0.062)	0.440
7y-baseline	0.138 (0.106, 0.170)	<0.001	0.132 (0.099, 0.164)	<0.001	0.006 (-0.040, 0.052)	0.790
8y-baseline	0.162 (0.128, 0.196)	<0.001	0.146 (0.112, 0.180)	<0.001	0.016 (-0.032, 0.064)	0.520

9y-baseline	0.143 (0.106, 0.180)	<0.001	0.173 (0.137, 0.209)	<0.001	-0.031 (-0.082, 0.021)	0.247
Lipid-lowering medication						
1y-baseline	-0.012 (-0.036, 0.012)	0.317	0.023 (0.002, 0.045)	0.033	-0.036 (-0.068, 0.003)	0.030
2y-baseline	-0.010 (-0.040, 0.019)	0.495	0.016 (-0.010, 0.042)	0.234	-0.026 (-0.066, 0.013)	0.193
3y-baseline	-0.001 (-0.033, 0.031)	0.973	0.044 (0.015, 0.073)	0.003	-0.045 (-0.088, 0.002)	0.042
4y-baseline	-0.004 (-0.038, 0.031)	0.842	0.023 (-0.008, 0.054)	0.146	-0.026 (-0.073, 0.020)	0.264
5y-baseline	-0.011 (-0.047, 0.024)	0.542	0.040 (0.009, 0.072)	0.013	-0.051 (-0.099, 0.004)	0.035
6y-baseline	-0.001 (-0.038, 0.035)	0.944	0.049 (0.016, 0.082)	0.003	-0.050 (-0.099, 0.001)	0.044
7y-baseline	0.020 (-0.017, 0.056)	0.291	0.062 (0.029, 0.094)	<0.001	-0.042 (-0.091, 0.007)	0.091
8y-baseline	0.034 (-0.006, 0.073)	0.093	0.090 (0.055, 0.125)	<0.001	-0.056 (-0.109, 0.003)	0.037
9y-baseline	0.052 (0.008, 0.097)	0.021	0.094 (0.056, 0.133)	<0.001	-0.042 (-0.101, 0.017)	0.164
Antithrombotics						
1y-baseline	0.047 (0.026, 0.068)	<0.001	0.031 (0.017, 0.046)	<0.001	0.016 (-0.010, 0.041)	0.231
2y-baseline	0.075 (0.051, 0.099)	<0.001	0.044 (0.027, 0.061)	<0.001	0.031 (0.002, 0.060)	0.038
3y-baseline	0.093 (0.066, 0.120)	<0.001	0.049 (0.031, 0.068)	<0.001	0.043 (0.011, 0.076)	0.009
4y-baseline	0.133 (0.103, 0.164)	<0.001	0.068 (0.047, 0.089)	<0.001	0.065 (0.028, 0.102)	0.001
5y-baseline	0.162 (0.128, 0.195)	<0.001	0.076 (0.054, 0.099)	<0.001	0.086 (0.045, 0.126)	<0.001
6y-baseline	0.189 (0.154, 0.225)	<0.001	0.094 (0.070, 0.119)	<0.001	0.095 (0.052, 0.138)	<0.001
7y-baseline	0.211 (0.173, 0.248)	<0.001	0.112 (0.085, 0.138)	<0.001	0.099 (0.053, 0.145)	<0.001
8y-baseline	0.235 (0.196, 0.275)	<0.001	0.135 (0.107, 0.164)	<0.001	0.100 (0.051, 0.149)	<0.001
9y-baseline	0.262 (0.217, 0.307)	<0.001	0.144 (0.113, 0.176)	<0.001	0.118 (0.063, 0.172)	<0.001
Glucose-lowering medication						
1y-baseline	0.012 (-0.000, 0.024)	0.051	0.013 (0.003, 0.022)	0.011	-0.001 (-0.016, 0.015)	0.922
2y-baseline	0.021 (0.005, 0.036)	0.008	0.023 (0.010, 0.036)	<0.001	-0.002 (-0.022, 0.018)	0.820
3y-baseline	0.026 (0.006, 0.045)	0.010	0.045 (0.028, 0.062)	<0.001	-0.019 (-0.045, 0.006)	0.141
4y-baseline	0.037 (0.016, 0.057)	<0.001	0.042 (0.025, 0.059)	<0.001	-0.005 (-0.032, 0.021)	0.692
5y-baseline	0.040 (0.018, 0.062)	<0.001	0.051 (0.033, 0.069)	<0.001	-0.011 (-0.039, 0.017)	0.438
6y-baseline	0.038 (0.014, 0.061)	0.002	0.061 (0.041, 0.081)	<0.001	-0.023 (-0.054, 0.007)	0.137
7y-baseline	0.049 (0.025, 0.074)	<0.001	0.061 (0.040, 0.081)	<0.001	-0.012 (-0.044, 0.021)	0.481
8y-baseline	0.063 (0.036, 0.089)	<0.001	0.073 (0.051, 0.096)	<0.001	-0.011 (-0.046, 0.024)	0.550
9y-baseline	0.062 (0.031, 0.093)	<0.001	0.082 (0.056, 0.108)	<0.001	-0.020 (-0.061, 0.020)	0.325
